# Presence of algal symbionts affects denitrifying bacterial communities in the sea anemone Aiptasia coral model

**DOI:** 10.1038/s43705-022-00190-9

**Published:** 2022-10-28

**Authors:** Nan Xiang, Nils Rädecker, Claudia Pogoreutz, Anny Cárdenas, Anders Meibom, Christian Wild, Astrid Gärdes, Christian R. Voolstra

**Affiliations:** 1 Marine Ecology Department, Faculty of Biology and Chemistry, University of Bremen, 28359 Bremen, Germany; 2 Alfred Wegener Institute, Helmholtz Centre for Polar and Marine Research, 27570 Bremerhaven, Germany; 3 Department of Biology, University of Konstanz, 78457 Konstanz, Germany; 4 Laboratory for Biological Geochemistry, School of Architecture, Civil and Environmental Engineering, École Polytechnique Fédérale de Lausanne (EPFL), 1015 Lausanne, Switzerland; 5 Center for Advanced Surface Analysis (CASA), Institute of Earth Science, University of Lausanne, 1015 Lausanne, Switzerland; 6 Hochschule Bremerhaven, Fachbereich 1, An der Karlstadt 8, 27568 Bremerhaven, Germany

**Keywords:** Microbial ecology, Biogeochemistry, Symbiosis

## Abstract

The coral-algal symbiosis is maintained by a constant and limited nitrogen availability in the holobiont. Denitrifiers, i.e., prokaryotes reducing nitrate/nitrite to dinitrogen, could contribute to maintaining the nitrogen limitation in the coral holobiont, however the effect of host and algal identity on their community is still unknown. Using the coral model Aiptasia, we quantified and characterized the denitrifier community in a full-factorial design combining two hosts (CC7 and H2) and two strains of algal symbionts of the family Symbiodiniaceae (SSA01 and SSB01). Strikingly, relative abundance of denitrifiers increased by up to 22-fold in photosymbiotic Aiptasia compared to their aposymbiotic (i.e., algal-depleted) counterparts. In line with this, while the denitrifier community in aposymbiotic Aiptasia was largely dominated by diet-associated *Halomonas*, we observed an increasing relative abundance of an unclassified bacterium in photosymbiotic CC7, and *Ketobacter* in photosymbiotic H2, respectively. Pronounced changes in denitrifier communities of Aiptasia with *Symbiodinium linucheae* strain SSA01 aligned with the higher photosynthetic carbon availability of these holobionts compared to Aiptasia with *Breviolum minutum* strain SSB01. Our results reveal that the presence of algal symbionts increases abundance and alters community structure of denitrifiers in Aiptasia. Thereby, patterns in denitrifier community likely reflect the nutritional status of aposymbiotic vs. symbiotic holobionts. Such a passive regulation of denitrifiers may contribute to maintaining the nitrogen limitation required for the functioning of the cnidarian-algal symbiosis.

## Introduction

Tropical coral reefs thrive in oligotrophic waters and are among the most diverse and productive ecosystems on Earth [1]. The efficient nutrient recycling in the coral holobiont, i.e., assemblages of coral host, intracellular algal symbionts of the family Symbiodiniaceae, and a diverse community of prokaryotic microorganisms, is the key to this ecological success [2–5]. In this photosymbiotic conglomerate, Symbiodiniaceae provide photosynthetically-fixed carbon to other holobiont members in exchange for carbon dioxide and inorganic nutrients from the coral host [6]. Importantly, the efficient carbon recycling in the holobiont is maintained by limited nitrogen availability for the algae [7]. A disruption of nitrogen limitation in the holobiont may promote uncontrolled growth and retention of photosynthates by algal symbionts [8, 9]. Thereby, the functioning of coral holobionts under anthropogenic change depends in part on their ability to maintain a nitrogen-limited state for the algal symbionts [10].

The taxonomically and functionally diverse prokaryotic microbiome associated with corals [11–15] contributes to holobiont nutrient cycling [16–19]. Diazotrophs, i.e., prokaryotes fixing atmospheric dinitrogen into ammonia, are ubiquitous members of coral holobionts [20–25]. Diazotrophs have been widely considered beneficial for corals on pristine oligotrophic reefs, as they supply ‘new’ nitrogen to help sustain coral productivity during low environmental nitrogen availability [19]. Conversely, stimulated diazotroph activity due to ocean warming and organic carbon eutrophication has been linked to the destabilization of the coral-algal symbiosis by disrupting nitrogen limitation in the holobiont [9, 21, 26]. In contrast, denitrifiers, i.e., prokaryotes that reduce nitrate/nitrite to dinitrogen [27], have been barely explored in the coral holobiont. However, denitrifiers could be key players in coral holobiont fitness and functioning, as they have the potential to help in maintaining nitrogen-limited conditions [10, 28–30].

Denitrifiers are ubiquitously associated with benthic reef organisms and likely show host-specific community compositions [28, 30]. A positive correlation of denitrifying activities in the coral holobiont with elevated nutrient concentrations in reef waters has been reported [31], which lends tentative support to the notion that denitrifiers help corals survive eutrophic conditions by maintaining nitrogen limitation within the holobiont [28–30]. Further, as denitrifying activity and Symbiodiniaceae density exhibit positive correlations in hard corals [28], it has been suggested that denitrifiers and Symbiodiniaceae may intimately interact within the coral holobiont, and that the former are limited by photosynthates released by the latter [28]. Indeed, denitrification is an energy-consuming process, and the denitrifying activity is typically limited by organic carbon availability [32]. Finally, a recent effort combining 16 S rRNA gene sequencing with functional inference suggests that a majority of denitrifiers in octocorals are members of the Symbiodiniaceae core microbiome [30]. Still, the factors driving denitrifier communities in the coral holobiont remain largely speculative; the effect of host and algal identity on the structuring of denitrifier communities remains unexplored.

Disentangling the respective contribution of host and algal symbionts to the structuring of prokaryotic communities is challenging in the intact coral holobiont, as it is difficult to maintain corals in aposymbiotic (i.e., algal-depleted) state and to manipulate their algal communities [33, 34]. In contrast, owing to the unique advantages of being amenable to manipulations of algal symbiont communities, the sea anemone *Exaiptasia diaphana* (from here on referred to as “Aiptasia”) is a model organism commonly used for functional studies on metabolic interactions in the coral-algal symbiosis, [35–37]. Here, we set out to assess the contribution of host and algal symbiont identity as drivers of denitrifier abundance and community composition in a full-factorial design, combining two Aiptasia strains in aposymbiotic state or symbiotic with two different Symbiodiniaceae clonal lineages (Supplementary Fig. S1). In addition, we assess the physiology and nutritional status of holobionts by measurements of symbiont density, photosynthetic efficiency, and carbon to nitrogen ratio. Thereby, we addressed the following questions: (1) Do denitrifier communities in Aiptasia differ between photosymbiotic states? (2) If so, does algal or host identity matter? And, finally: (3) Do patterns in denitrifier abundance and community composition reflect the nutritional status of holobionts?

## Materials and methods

### Aiptasia rearing, experimental design, and sampling

Anemones of the clonal anemone *Exaiptasia diaphana* (‘Aiptasia’) strains CC7 (North Carolina) [38] and H2 (Hawaii) [39] were kept in separate 3 L containers filled with artificial seawater (salinity 35 PSU; Pro Reef, Tropic Marine, Germany) at 25 °C with a 12 h:12 h light/dark cycle at 60 μE m^−2^ s^−1^. Aiptasia were fed with freshly prepared brine shrimp (*Artemia salina*) nauplii once a week, followed by tank cleaning and full water exchange the next day. The two Aiptasia strains CC7 and H2 are genetically distinct and differ in their native dominant endosymbiont [40]. Aiptasia CC7 is natively associated with the symbiont SSA01, a strain of *Symbiodinium linucheae* [38, 41]. The Aiptasia H2 is natively associated with the symbiont SSB01, a strain of *Breviolum minutum* [39, 42]. To verify the stability of native Symbiodiniaceae communities in Aiptasia cultures, symbiont identity was regularly confirmed using genus-specific primers [43] (see the following qPCR methodology section for details). Briefly, photosymbiotic Aiptasia were depleted of their native symbiont by incubation in 4 °C seawater for 4 h, followed by 2 days at 25 °C in artificial seawater containing the photosynthesis inhibitor diuron as outlined previously (Pringle lab 2018. Cold-shock protocol to bleach Aiptasia. protocols.io 10.17504/protocols.io.qx8dxrw). The aposymbiotic Aiptasia were maintained in the dark for more than 6 months. Prior to experiments, the absence of symbiont was confirmed via a lack of chlorophyll autofluorescence, assessed by fluorescence stereomicroscopy. For inoculations of Aiptasia with algal symbionts, the aposymbiotic animals were incubated twice in artificial seawater containing 10^5^ Symbiodiniaceae cells mL^−1^ during the feeding with *Artemia salina* nauplii in 2 consecutive weeks. Thereby, our experimental design generated six distinct host-symbiont combinations of Aiptasia: aposymbiotic CC7 (CC7-APO), CC7 with symbiont SSA01 (CC7-SSA01), CC7 with symbiont SSB01 (CC7-SSB01); aposymbiotic H2 (H2-APO), H2 with symbiont SSA01 (H2-SSA01), H2 with symbiont SSB01 (H2-SSB01) (for a detailed overview of the experimental setup, refer to Supplementary Fig. S1). Six host-symbiont combinations were maintained in different tanks containing ten animals each. This allowed us to disentangle the contribution of host and algal identity to the structuring of denitrifier communities in three comparisons: (a) between aposymbiotic and photosymbiotic states of the same host, (b) between different symbionts within the same host, (c) between different hosts with the same symbiont. For Aiptasia sampling, five replicates were collected for each group, in total 5 replicates * 6 groups = 30 samples. To minimize effects of *Artemia* feeding, we cleaned the Aiptasia animals the day after feeding and sampled them 2 days after cleaning.

### Assessment of host tissue protein content and Symbiodiniaceae cell density

Each Aiptasia was homogenized with 500 µl filtered (0.22 µm) 2× phosphate buffered saline (PBS) in a 1.5 ml sterile Eppendorf tube using a cordless Pellet Pestle^™^ (Fisherbrand, USA). For the measurement of host protein content, 100 µl homogenate aliquots were centrifuged at 3000 × *g* for 3 min using Centrifuge 5910 R (Eppendorf, Germany). For each sample, three technical replicates of 5 µl of the five-fold diluted supernatant were transferred into a 96-well plate. The protein content was quantified using the Pierce^™^ Coomassie Plus (Bradford) Assay Kit (Thermo Scientific, US) following the manufacturer’s protocol and recording the absorbance at 595 nm using a CLARIOstar Plus plate reader (BMG LABTECH, Germany). For Symbiodiniaceae density, 100 µl aliquots of homogenate were centrifuged at 3000 × *g* for 3 min. 500 µl filtered 2× PBS was added to resuspend the Symbiodiniaceae pellets. The Symbiodiniaceae density was quantified for six technical replicate aliquots for each Aiptasia sample using a Countess II FL (Invitrogen, USA) fluorescence cell counter reading from channel CY5.

### Photosynthetic efficiency and analysis of elemental carbon and nitrogen

The maximum photosynthetic efficiency of six host-symbiont combinations was assessed *via* pulse-amplitude modulation (PAM) fluorometry. Aiptasia were dark-adapted for 30 min and the ratio between variable and maximum chlorophyll fluorescence (F_v_/F_m_) was measured using the blue version of the MINI-PAM-II (Walz, Germany) with a 5.5 mm fiber optic. For measuring the elemental carbon and nitrogen contents, Aiptasia animals were collected and homogenized with 500 µl filtered 2× PBS in a 1.5 ml sterile Eppendorf tube. Homogenized tissues were transferred on a clean 0.22 µm filter and dried at 40 °C until constant weight. Around 5 mg of dried sample was used to measure total carbon to nitrogen ratio (C:N ratio) using a Euro EA-CHNSO Elemental Analyzer (HEKAtech, Germany).

### DNA extraction, *nirS* amplification, and quantitative PCR (qPCR)

Genomic DNA of samples (including three negative extractions) were extracted with the DNeasy Blood and Tissue Kit according to manufacturer instructions (Qiagen, Germany). Extracted DNA was quality-checked by spectrophotometry at 260 nm and 280 nm on a Nanodrop 2000C spectrophotometer (Thermo Scientific, USA), and quantified using a Qubit and the dsDNA High Sensitivity Assay Kit (Invitrogen, USA).

The relative abundance of Symbiodiniaceae strains in symbiotic animals was quantified by qPCR with genus-specific primer pairs using a qTOWER3 84 G (Analytik Jena, Germany) with the innuMIX qPCR DSGreen Standard kit (Analytik Jena, Germany). Each qPCR reaction consisted of: 5 μl of MasterMix, 0.5 μl of 10 μM forward and reverse primer each, 3 μl of nuclease-free water, and 1 μl DNA template (10 ng µl^−1^) for a total reaction volume of 10 μl. The thermal cycling condition consisted of a hot-start activation at 95 °C for 15 min, followed by 40 cycles of denaturation at 95 °C for 30 s, annealing at 60 °C for 30 s, and extension at 72 °C for 30 s. Final extension was carried out at 72 °C for 10 s followed by a melting curve from 65 °C to 95 °C with an increase of 0.5 °C every 5 s. Samples were amplified using the *Symbiodinium-*specific primer pair A-F (5′-CCTCTTGGACCTTCCACAAC-3′) and A-R (5′-GCATGCAGCAACACTGCTC-3′) as well as the *Breviolum*-specific primer pair B-F (5′-GTCTTTGTGAGCCTTGAGC-3′) and B-R (5′-GCACACTAACAAGTGTACCATG-3′) [43]. To obtain the relative proportions of these two Symbiodiniaceae genera (clades) in each sample, the ∆Ct for each sample was calculated based on the cycle threshold (Ct) values of Symbiodiniaceae SSA01 against Ct values of SSB01, followed by a reference against the ∆Ct of DNA extracted from a 1:1 ratio of SSA01 and SSB01 cells using the ∆∆Ct method [44]. The qPCR efficiency for *Symbiodinium-*specific and *Breviolum*-specific amplifications were 95.28% and 97.45%, respectively.

The nitrite reductase *nirS* gene was used as a marker to quantify and characterize the denitrifier community [27]. Following the same reagent conditions for qPCR reactions as outlined above, samples were amplified by the thermal cycling condition consisted of a hot-start activation at 95 °C for 15 min, followed by 40 cycles of denaturation at 95 °C for 30 s, annealing at 65 °C (*nirS*) for 30 s and extension at 72 °C for 30 s. Final extension was carried out at 72 °C for 10 s followed by a melting curve from 65 °C to 95 °C with an increase of 0.5 °C every 5 s. A fraction of the *nirS* gene was amplified using the primer pair nirS-1F (5′-CCTAYTGGCCGCCRCART-3′) and nirS-qR (5′-TCCMAGCCRCCRTCRTGCAG-3′) [45] and the specificity of amplification was confirmed with Sanger sequencing (Eurofins Genomics, Germany). Relative fold change of the *nirS* gene was referenced to the mean Ct of aposymbiotic CC7 by ∆Ct method with normalized genomic DNA quantity of the whole Aiptasia holobiont [46]. The qPCR efficiency for *nirS* gene amplification was 100.8%.

### *nirS* gene amplicon library preparation and sequencing

To characterize the denitrifier community composition, the *nirS* primer pair 1 F, qR (previously used for qPCR as outlined above) with sample-specific paired NovaSeq barcodes was used to amplify a region of the *nirS* gene. PCR was run using a reaction volume of 10 μl containing 5 μl of Qiagen multiplex PCR master mix (Qiagen, Germany), 1 μl of 5 μM forward and reverse primer each, 2 μl nuclease-free water, and 1 μl DNA template (10 ng µl^−1^). Thermal cycling condition consisted of an initial activation at 95 °C for 15 min, followed by 40 cycles of 95 °C for 10 s, 65 °C for 20 s, and 72 °C for 30 s, followed by a final extension step at 72 °C for 10 min. The PCR products were quality-checked on a 1% gel via electrophoresis using EPS 150/2000 (VWR, USA) and purified using one-Step ExoProStar (Fisher Scientific, USA). Purified PCR products were indexed using the Nextera XT Index Kit v2 (Illumina, USA) according to manufacturer instructions. Indexed samples were cleaned and normalized using the SequalPrep Normalization Plate Kit (Invitrogen, USA). All samples were pooled in one 1.5 ml Eppendorf tube and concentrated to 100 µl using a Vacuum Concentrator plus (Eppendorf, Germany). The final DNA yield of pooled 48 samples (distributed over 34 Aiptasia animals in 6 distinct host-symbiont combinations, 3 SSA01 algal culture aliquots, 3 SSB01 algal culture aliquots, 3 Artemia culture aliquots, 3 negative control DNA extractions, 2 negative control PCR reactions) was 414 ng determined by Qubit dsDNA High Sensitivity Assay (Invitrogen, USA). Sequencing was conducted by Novogene (Cambridge, UK) on the NovaSeq 6000 PE250 platform (Illumina, USA) using 2*250 bp paired-end reads.

### Sequence processing and analysis

For *nirS* amplicons, sequence quality control was conducted in R (version 4.1.0) following the DADA2 Pipeline Workflow (version 1.22.0) with slight modifications [47]. Primers were removed from the demultiplexed sequences with “Cutadapt” v.2.10 [48]. Due to the biological length variation of *nirS* gene [27], the paired-end reads were quality filtered by the minimum length of 100 bp, as determined by the length distribution and identity check. Reads were de-replicated, error rates were estimated and used for inference of true amplicon sequence variants (ASV). After merging paired reads, a *nirS* ASV table was constructed with chimera removal by the *de novo* approach. ASVs were filtered by hits of gene “*nirS*” after Blastx against the UniprotKB/Swiss-Prot database [49]. Remaining ASVs were filtered by the length of 220–240 bp, as determined by the length distribution, and were further translated to the protein level in a correct open reading frame using TranslatorX [50] and ORFfinder in NCBI. The *nirS* protein sequences were aligned by multiple alignment in MUSCLE 3.8.425 [51]. A *nirS* phylogenetic tree was built using Geneious Tree Builder in Geneious Prime (version 2021.0.3) [52]. For taxonomic assignment, a protein sequence database of the *nirS* gene was downloaded from the FunGene repository [53] (accessed 10/01/2022). Sequences belonging to uncultured organisms were excluded, which resulted in a customized FunGene database containing 3,504 *nirS* gene sequences with known taxonomy across 238 genera and 894 species. Query *nirS* protein sequences were run with blastp against the customized FunGene database for the taxonomic assignment. Determined *nirS* sequencing data are available on NCBI under BioProject PRJNA836569. All bioinformatic workflows can be accessed at: https://github.com/NancyXiang/nirS-Sequence-Analysis.

### Statistical analysis

Data analysis and plotting were conducted in R (version 4.1.0) using several packages, such as “ggplot2” [54] and “vegan” [55]. After log transformation to meet data normality, symbiont density, C:N ratio, and photosynthetic efficiency were analyzed by two-way analysis of variance (ANOVA) defining host strains (2 levels: CC7 and H2) and photosymbiotic associations (3 levels: aposymbiotic, SSA01 photosymbiotic and SSB01 photosymbiotic) as two factors followed by Tukey’s honestly significant difference (HSD) as a post hoc comparison. For non-normally distributed qPCR data, Kruskal–Wallis were used with Dunn test as post hoc comparisons. For *nirS* sequencing data, ASVs were considered putative contaminants if their relative abundances were >10% in negative controls. Alpha diversity was calculated based on repeated random subsampling of ASVs to the minimum library size of 12,521 reads. Statistical differences were tested for each Alpha diversity index using a two-way ANOVA with Tukey’s HSD. Beta diversity was calculated using Euclidean distances of centered log-ratio (clr)-transformed ASV counts. Statistical differences in beta diversity between host strains and across photosymbiotic associations were tested using Permutational multivariate analysis of variance (PERMANOVA, ‘adonis’ function, 999 permutations) and represented on a Principal component analysis (PCA) ordination using Phyloseq v1.38.0 [56]. Analysis of Compositions of Microbiomes with Bias Correction (ANCOM-BC) [57] was used to identify differentially abundant ASVs between host strains and between photosymbiotic associations within each host.

## Results

### Algal depletion and algal inoculation in Aiptasia

**Fig. 1:**
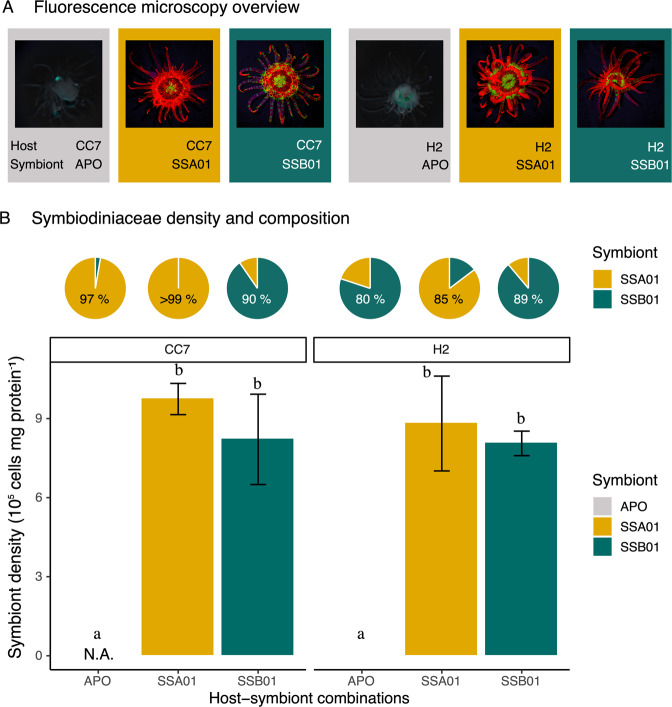
Inoculations of aposymbiotic Aiptasia with algal symbionts. **A** Fluorescence microscopy overview of six host-symbiont combinations to visualize *in hospite* chlorophyll of photosymbiotic Symbiodiniaceae. **B** Symbiodiniaceae density and community composition of six host-symbiont combinations. All data are shown as mean ± SE (*n* = 5 animals each). Different letters above bars indicate significant differences between groups (two-way ANOVA with Tukey HSD, *P* < 0.05).

Inoculations of two aposymbiotic Aiptasia strains with two different algal strains were confirmed using fluorescence microscopy (Fig. 1A) and counts of algal density in host tissues (Fig. 1B). The algal density significantly differed between host strains (Fig. 1B; two-way ANOVA, F_1,16_, *P* = 0.001) and across photosymbiotic associations (Fig. 1B; two-way ANOVA, F_2,16_, *P* < 0.001). While aposymbiotic CC7 showed no detectable algae by fluorescence cell counter, genus-specific qPCR identified a background population of algal symbionts dominated by *Symbiodinium linucheae* strain SSA01, making up 97.3% of the residual Symbiodiniaceae community (composed of *Symbiodinium* and *Breviolum*). Likewise, a low number of algal symbionts (0.004 ± 0.004 cells mg-1 host protein) were detected in aposymbiotic H2, with *Breviolum minutum* strain SSB01 dominating 80.1% of the residual Symbiodiniaceae community (composed of *Symbiodinium* and *Breviolum*, Fig. 1B). Aiptasia with algal symbionts had significantly higher algal densities than their aposymbiotic counterparts (Fig. 1B; Tukey HSD of CC7, *P* < 0.001; Tukey HSD of H2, *P* < 0.001). Aiptasia CC7 and H2 accommodated non-significantly higher algal densities per mg host protein when associated with symbiont SSA01, compared to when associated with symbiont SSB01 (Fig. 1B; Tukey HSD, *P* = 0.856 and *P* = 0.998, respectively). In Aiptasia with algal symbionts, the respective algal strain used for inoculations dominated the Symbiodiniaceae community of CC7 and H2 (SSA01: > 99.99 and 85.32%, respectively; SSB01: 90.73 and 88.84%, respectively).

### High carbon availability in Aiptasia with algal symbiont SSA01 aligns with photo-physiological performance

**Fig. 2:**
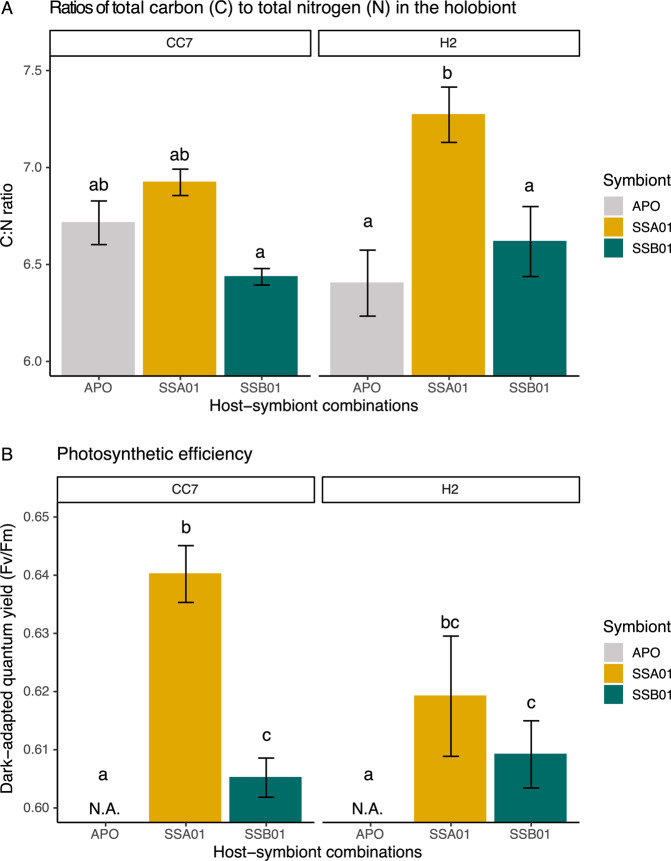
Physiology of six host-symbiont combinations of Aiptasia. **A** Ratios of total carbon to total nitrogen. **B** Photosynthetic efficiency of photosystem II. All data are presented as mean ± SE (*n* = 5 animals each). Different letters above bars indicate significant differences between groups (two-way ANOVA with Tukey HSD, *P* < 0.05).

Aiptasia with SSA01 showed significantly higher C:N ratios than their SSB01 photosymbiotic or aposymbiotic counterparts (Fig. 2A; two-way ANOVA, F_2,24_, *P* < 0.001), regardless of the host identity (two-way ANOVA, F_1,24_, *P* = 0.585). Aiptasia CC7 showed a fairly stable C:N ratio across different photosymbiotic associations, i.e., homologous SSA01 symbiotic, heterologous SSB01 symbiotic, and aposymbiotic (Tukey HSD, *P* = 0.129–0.869). In contrast, Aiptasia H2 with heterologous symbiont SSA01 showed ~50% and ~70% higher C:N ratio compared to those with homologous symbiont SSB01 (Tukey HSD, *P* = 0.024) and their aposymbiotic counterparts (Tukey HSD, *P* = 0.001), respectively. Higher photosynthetic efficiency was found in Aiptasia with symbiont SSA01 than those with SSB01 (Fig. 2B; two-way ANOVA, F_1,16_, *P* = 0.004; Tukey HSD of CC7, *P* = 0.009; Tukey HSD of H2, *P* = 0.723).

### Higher denitrifier abundance in photosymbiotic Aiptasia in comparison to aposymbiotic counterparts

**Fig. 3:**
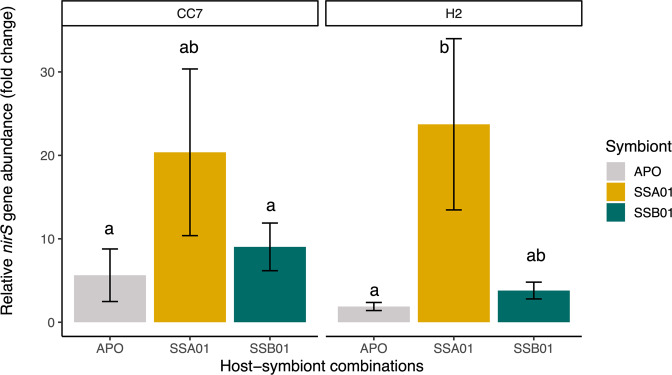
Relative abundance of denitrifiers. Relative fold change in copy numbers of the *nirS* gene in relation to the mean Ct of aposymbiotic CC7 samples. DNA inputs for qPCR are normalized. All data are presented as mean ± SE (*n* = 5 animals each). Different letters above bars indicate significant differences between groups (Kruskal–Wallis with Dunn test, *P* < 0.05).

Relative *nirS* gene copy numbers, i.e., relative abundances of denitrifiers quantified by *nirS* qPCR did not differ between host strains (Fig. 3; Kruskal–Wallis, *P* = 0.438), but significantly differed across photosymbiotic associations (Fig. 3; Kruskal–Wallis, *P* = 0.006). The relative abundance of denitrifiers in Aiptasia with symbiont SSA01 were nearly 4- to 23-fold higher compared to their SSB01 photosymbiotic (Fig. 3; Kruskal–Wallis, *P* = 0.135) and aposymbiotic counterparts (Fig. 3; Kruskal–Wallis, *P* = 0.004).

### *nirS* sequencing overview

After demultiplexing samples and processing raw reads with the DADA2 pipeline, we obtained 1104 ASVs represented by 7,414,102 reads. We retained 616 ASVs (represented by 6,907,743 reads) after confirming their functional annotation to *nirS* by Blastx against SwissProt, from which we kept 565 ASVs represented by 6,907,542 reads that had a length of 220–240 bp. Of these, 558 ASVs represented by 6,905,196 reads were successfully translated to *nirS* proteins in a correct open reading frame. After discarding ASVs considered as contaminants, 427 ASVs represented by 5,378,208 reads distributed over 48 samples were retained for downstream statistical analysis and visualization. Of these, 305 ASVs (3,867,048 reads) were assigned to a taxonomic group as opposed to 122 ASVs which could not be classified. A total of 27 genera of denitrifiers were identified across 43 samples (including 34 host-symbiont combinations of Aiptasia animals, 6 algal culture samples, and 3 food samples). The sequencing data were highly dominated by a few abundant ASVs, three dominant taxa *Halomonas* (ASV1; Gammaproteobacteria, *Halomonadaceae*), an unclassified bacterial symbiont of *Nonionella stella* (ASV2) and *Ketobacter* (ASV3; Gammaproteobacteria, *Alcanivoracaceae*) made up to 83.06% of the total sequencing reads (33.44%, 26.64%, and 22.98%, respectively). The alpha diversity indices showed no significant difference between host strains (Supplementary Fig. S2; Chao1 richness: *F*_1,26_ = 0.004, *P* = 0.952; Shannon diversity: *F*_1,26_ = 0.508; *P* = 0.482; Simpson evenness: *F*_1,26_ = 0.292, *P* = 0.594) or across photosymbiotic associations (Chao1 richness: *F*_2,26_ = 1.351, *P* = 0.277; Shannon diversity: *F*_2,26_ = 0.355; *P* = 0.704; Simpson evenness: *F*_2,26_ = 0.323; *P* = 0.727).

### Pronounced shift of denitrifier community composition aligns with algal photo-physiology

**Fig. 4:**
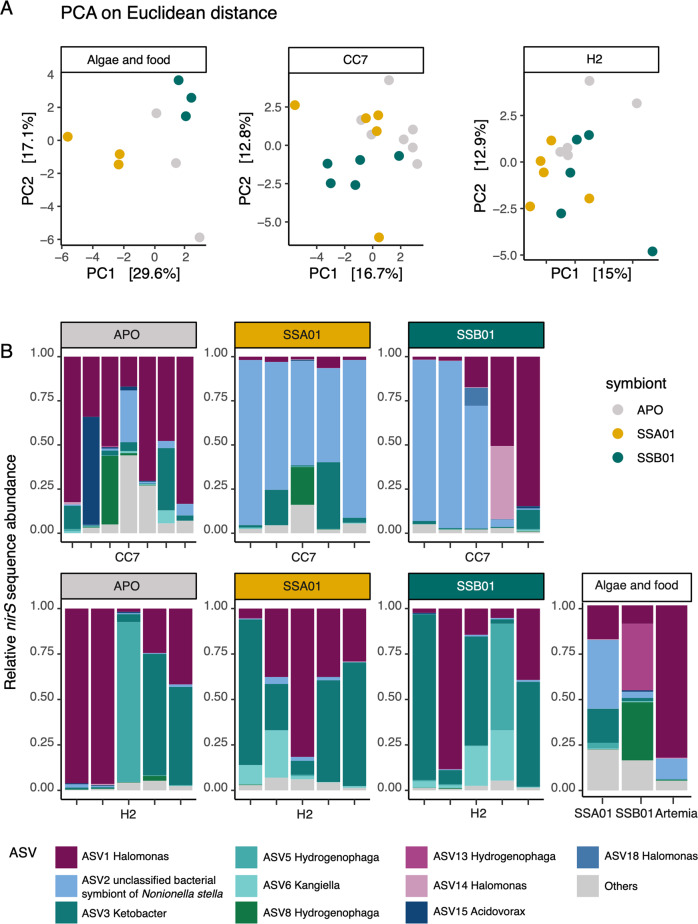
Denitrifier community composition based on *nirS* gene sequencing. **A** Principal component analysis (PCA) plots of denitrifier community compositions associated with Aiptasia, algal cultures, and food Artemia based on Euclidean distances. **B** Stacked bar plots showing denitrifier community compositions in Aiptasia, algal cultures, and food Artemia. Stacked bar plots display the 10 most abundant bacterial amplicon sequence variants (ASVs).

**Fig. 5:**
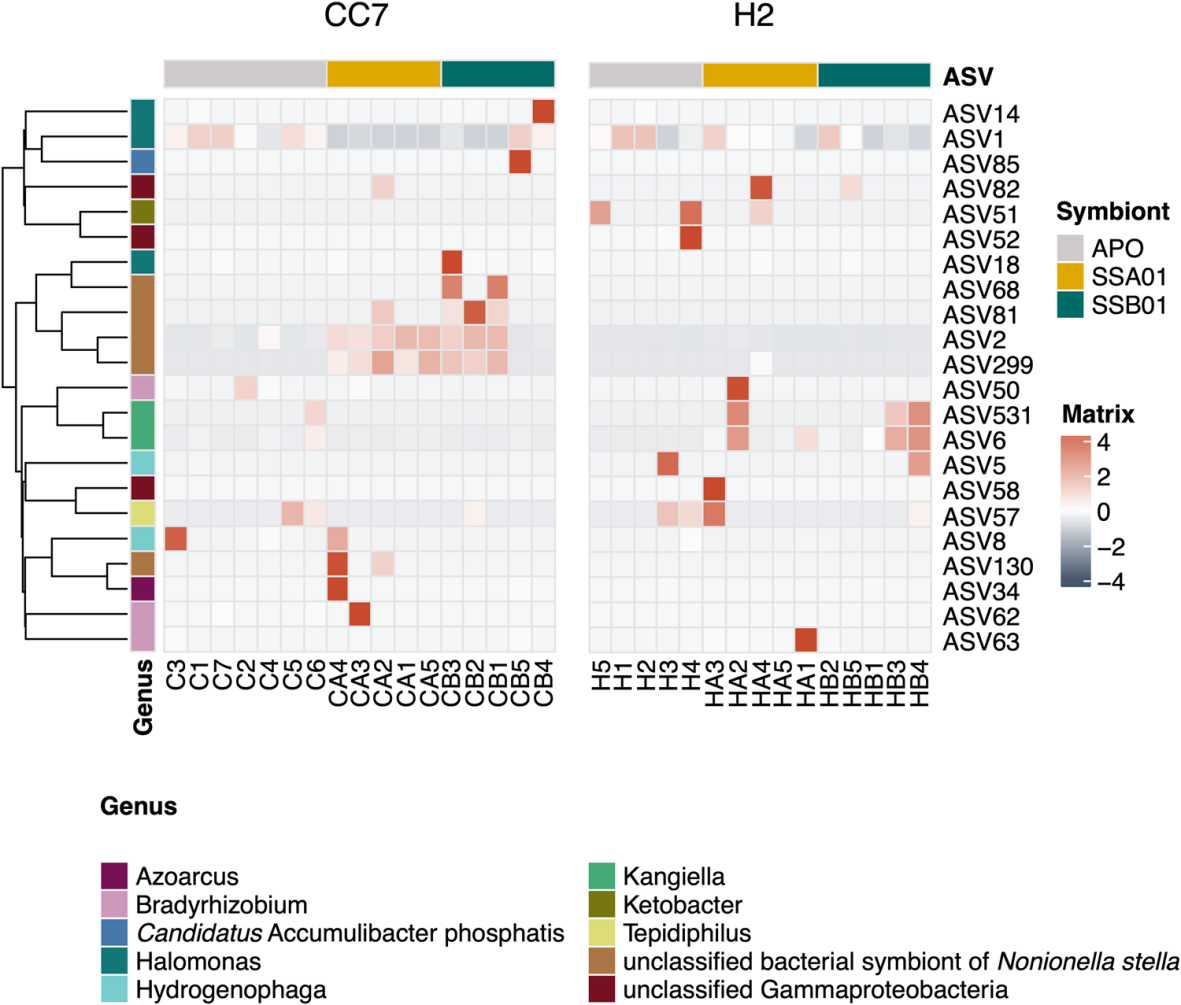
Differential enrichment of denitrifier taxa across six host-symbiont combinations of Aiptasia. Heatmap showing the significantly enriched amplicon sequence variants (ASVs) across samples.

The denitrifier community compositions were distinct among Aiptasia animals, algal culture samples, and food samples (Fig. 4A, B; PERMANOVA, *F*_2,38_ = 1.31, *P* = 0.045). The predominant ASV1 (Fig. 5; Gammaproteobacteria, *Halomonadaceae*, *Halomonas*) in food samples (~80% of the total sequences) showed its prevalence across the entire dataset (Fig. 4B). Focusing on Aiptasia, their denitrifier community composition was affected by both host identity (Fig. 4A, B; PERMANOVA, *F*_1,30_ = 2.20, *P* = 0.001) and photosymbiotic association (Fig. 4A, B; PERMANOVA, *F*_2,29_ = 1.27, *P* = 0.038).

Denitrifier communities in aposymbiotic CC7 and H2 showed no significant difference and were both highly dominated by ASV1 (Fig. 4B; PERMANOVA, *F*_1,10_ = 1.00, *P* = 0.473). After inoculations with algal symbionts, the denitrifier community compositions varied considerably in both Aiptasia strains (Fig. 5; PERMANOVA of CC7: *F*_2,14_ = 1.41, *P* = 0.002; PERMANOVA of H2: *F*_2,12_ = 1.34, *P* = 0.024). The post hoc analysis identified significant differences between Aiptasia with symbiont SSA01 and their aposymbiotic counterparts (Fig. 5; PERMANOVA pairwise of CC7: *F* = 1.83, *P* = 0.003; PERMANOVA pairwise of H2: *F* = 1.62, *P* = 0.048). The effects of symbiont SSA01 on denitrifier community structure showed distinct manners between host strains (Fig. 4A, B; PERMANOVA pairwise, *P* = 0.002). In Aiptasia CC7 with symbiont SSA01, it was reflected in the enrichment of the predominant taxon ‘unclassified bacterial symbiont of *Nonionella stella*’ (Fig. 5; ASV2, ASV130, ASV299). In contrast, in Aiptasia H2 with symbiont SSA01, this difference was driven by diverse enriched taxa (Fig. 5), such as ASV6 (Gammaproteobacteria, *Kangiellaceae*, *Kangiella*), ASV50 and ASV63 (Alphaproteobacteria, *Nitrobacteraceae*, *Bradyrhizobium)*, and ASV58 (unclassified Gammaproteobacteria).

## Discussion

Nitrogen cycling microbes are considered key players in the coral holobiont due to their potential role in stabilizing or destabilizing nitrogen limitation, critical for the functioning of the coral-algal symbiosis [10, 16, 17, 21, 26]. To disentangle the putative direct or indirect contribution of the cnidarian host and algal symbionts in the structuring of denitrifier communities, here we quantified the abundance and characterized the community composition of denitrifiers in two Aiptasia strains in an aposymbiotic or symbiotic state with two different Symbiodiniaceae clonal lineages. We found that the presence of algal symbionts strongly increased the denitrifier abundance in Aiptasia holobionts, while the denitrifier community composition was jointly affected by both host and algal identity. As denitrifier abundance aligned with the photosynthetic efficiency of algal symbionts and holobiont-associated C:N ratios, our findings suggest that denitrifier communities are passively regulated by the nutritional status of holobionts and thereby contribute to maintaining a nitrogen-limited state.

### Diet-derived denitrifiers dominate *nirS* communities of aposymbiotic Aiptasia

To the best of our knowledge, this study is the first next-generation sequencing characterization of the *nirS*-based denitrifier communities in a photosymbiotic cnidarian. Our results reveal that Aiptasia harbors distinct denitrifier communities with low phylogenetic diversity, dominated by a few bacterial taxa affiliated to Alpha- and Gammaproteobacteria. Thereby, our findings resemble patterns previously reported for diazotroph community compositions in corals, characterized by a high dominance of only a few taxa and similar taxonomy at the Class level [9, 58, 59].

Similar to previous reports [60], artificially bleached Aiptasia still harbored a background community of algal symbionts. However, the low symbiont densities in these animals (orders of magnitude lower than in fully photosymbiotic animals) and the successful replacement of the dominant algal symbionts in symbiotic animals suggest that these animals can be considered functionally aposymbiotic for the purpose of this study. Previous studies reported high rates of inorganic nitrogen release in aposymbiotic Aiptasia due to host catabolic activity [36]. While previous studies suggested that these conditions might favor the growth of denitrifiers [61], aposymbiotic Aiptasia showed the lowest relative abundance of denitrifiers among six different host-symbiont combinations in the present study. This finding is further supported by the strong dominance of ASV1 *Halomonas* in denitrifier communities of aposymbiotic Aiptasia from both clonal lineages. As ASV1 accounted for ~80% of *nirS* sequences associated with *Artemia* nauplii used for feeding of the animals, the dominance of this ASV in denitrifier communities thus likely reflects the ingestion (and presumably, digestion) of these microbes by the host rather than being a stable component of these animal microbiomes [35, 62]. Coupled with the absence of host lineage-specific community differences in aposymbiotic animals, our results suggest that denitrifier communities are extremely rare in the aposymbiotic Aiptasia holobiont and largely reflect the opportunistic acquisition and diet by the host. The low denitrifier abundances imply reduced microbial nitrogen transformation processes in aposymbiotic Aiptasia [35]. Consequently, our findings suggest that denitrifiers could not play an important role in the nitrogen cycle of aposymbiotic cnidarian holobionts.

### Presence of algal symbionts promotes denitrifier abundance in photosymbiotic Aiptasia

Increased denitrifier abundances in photosymbiotic Aiptasia compared to their aposymbiotic counterparts were accompanied by altered denitrifier community structures. These findings align with the relative depletion of sequences affiliated to ASV1 *Halomonas* (presumably diet-derived) in photosymbiotic animals, indicating that the denitrifier proliferation may be driven by increased Symbiodiniaceae densities. Interestingly, the changes in denitrifier abundance and community composition showed clear symbiont-specific patterns. Aiptasia with symbiont SSA01 not only exhibited the highest relative abundances of denitrifiers, but also harbored more differently enriched denitrifier taxa compared to their aposymbiotic or SSB01 symbiotic counterparts. Specifically, Aiptasia with symbiont SSA01 were enriched in denitrifiers assigned as unclassified bacterial symbiont of the foraminifera *Nonionella stella* (consisting of ASV2 and ASV299). As these denitrifiers also dominated the *nirS* community of SSA01 algal cultures, our results suggest that the denitrifier community shifts observed here may, in part, reflect the colonization of the holobiont by algal-associated bacteria [63, 64]. In line with this, Aiptasia with symbiont SSB01 showed similar, albeit less pronounced, increases in denitrifier abundance and specific enrichment of rare (i.e., low abundant) denitrifying taxa (e.g., ASV85 *Candidatus* Accumulibacter phosphatis) compared to aposymbiotic animals.

While the presence of algal symbionts caused pronounced shifts in denitrifier abundances and community compositions of Aiptasia, patterns of community change also depended on the host identity. The denitrifier community in strain CC7 with symbiont SSA01 was dominated by the unclassified bacterial symbiont of *Nonionella stella* (ASV2, ASV130, ASV299) compared to their aposymbiotic counterparts. In contrast, Aiptasia H2 with symbiont SSA01 was enriched in *Kangiella* (ASV6, ASV58) and *Bradyrhizobium* (ASV50, ASV63) compared to aposymbiotic counterparts. It is thus evident that the proliferation of denitrifiers following increased Symbiodiniaceae densities either favors different algal-associated bacteria depending on host identity or that rare denitrifiers present in aposymbiotic hosts colonize the holobiont in an opportunistic manner. The functional importance of the distinct bacterial community between photosymbiotic and aposymbiotic Aiptasia is currently unclear [61, 65]. Here, our findings suggest that the functional importance of denitrifiers in nutrient cycling is limited to the photosymbiotic holobiont, and their community structures depend on both host and symbiont types.

### Denitrifier communities reflect the nutritional status of holobionts

There are many environmental drivers that affect denitrifying bacterial activity, such as nitrogen, oxygen, and organic carbon availability [32]. However, the abiotic and biotic drivers of the denitrifier community in the cnidarian holobiont remain unknown. Previous studies have shown that increased algal densities enhanced daytime oxygen availability and nitrogen competition within the Aiptasia holobiont [35, 36]. To this end, our findings suggest that the availability of oxygen and nitrogen are not major driving forces for denitrifier communities in the present study. The symbiosis of cnidarian host and algal symbionts and concomitant increase in photosynthate availability are known to drastically alter the nutrient availability in the cnidarian holobiont [35, 36]. Specifically, the efficient recycling of organic and inorganic carbon in the symbiosis causes a proportional reduction in nitrogen availability in the photosymbiotic holobiont [35, 36]. The reduced nitrogen and increased oxygen (under light conditions) availability in photosymbiotic holobionts may indicate unfavorable conditions for denitrifier communities, as denitrification is an anaerobic process [32]. However, we observed a pronounced proliferation of denitrifiers following the increase in Symbiodiniaceae densities. Interestingly, a majority of denitrifiers detected in photosymbiotic Aiptasia (e.g., *Kangiella*, *Bradyrhizobium* and *Candidatus* Accumulibacter phosphatis) are heterotrophic bacteria [66–68]. In line with this, previous studies suggested that the release of photosynthetically-fixed carbon from Symbiodiniaceae promotes bacterial denitrification in the cnidarian holobiont [28].

Importantly, the presence of symbiont SSA01 caused the most pronounced effects on denitrifier communities in both Aiptasia CC7 and H2 holobionts. Concurrently, SSA01-symbiotic holobionts showed consistently higher photosynthetic efficiency and symbiont densities than their SSB01-symbiotic counterparts. Coupled with the higher C:N ratios in SSA01-symbiotic holobionts, our results suggest that SSA01 shows superior physiological performance (approximated via the assessment of photosynthetic efficiency by PAM fluorometry) *in hospite* and causes a more pronounced alteration in the nutrient cycling of Aiptasia holobionts. It is thus plausible that different patterns in denitrifiers in the present study largely reflect the opportunistic growth of bacteria in response to the enhanced carbon availability of photosymbiotic holobionts. In this scenario, the specific effect of host and symbiont identity on denitrifier communities thereby reflects the nutritional consequences of distinct photo-physiological performance of respective host-algal symbiont combinations [36, 69, 70].

Notably, the present study was carried out under controlled laboratory conditions with a limited reservoir of environmental microbes. Denitrifier communities in natural populations of Aiptasia thus likely differ in that they may be more complex and dynamic than those investigated here. However, the proposed link between the nutritional state of holobionts and denitrifier communities implies that the described patterns in abundance and community diversity are similar in more complex natural denitrifier communities of cnidarian holobionts.

Patterns in the denitrifier community (and by extension, denitrification activity) as consequences of holobiont carbon availability have important implications for the functioning of cnidarian-algal symbiosis. In this scenario, abundant algal photosynthate release might indirectly promote the opportunistic growth of heterotrophic denitrifiers. Resulting increases in denitrifier activity would, in turn, limit holobiont nitrate/nitrite availability for algal symbionts, thereby stabilizing the nitrogen-limited conditions required for efficient carbon recycling in the holobiont [7, 8, 26]. This positive feedback loop between photosynthate release and denitrification effectively contributes to maintaining the cnidarian-algal symbiosis in a stable holobiont state. At the same time, such dependence of denitrifiers on organic carbon availability may limit their potential contribution to holobiont acclimatization and adaptation [5, 15]. As decreases in algal photosynthate release under ocean warming [8] would directly reduce denitrifier abundance (hence, denitrification activity) [28], denitrifiers may not effectively contribute to maintaining nitrogen limitation at ecologically and physiologically relevant scales in a disturbed cnidarian holobiont.

## Conclusion

The global decline of coral reefs urges the need for a better understanding of holobiont functioning and composition. Here we show that the increased Symbiodiniaceae density alters *nirS* denitrifier abundance and community composition in the Aiptasia holobiont. Changing patterns in denitrifier communities align with the physiological performance of algal symbionts within their respective hosts. As such, denitrifier community patterns likely reflect the nutritional status of holobionts. Passively regulated by organic carbon availability, denitrifiers could effectively contribute to maintaining the nitrogen limitation characteristic of a stable cnidarian holobiont. Future metagenomic and metatranscriptomic studies between photosymbiotic and aposymbiotic Aiptasia, in conjunction with culture-dependent microbial work targeting denitrifiers may provide new insights into the functional attributes of holobiont-associated microbial communities.

## Supplementary Information

Supplementary Information

## Data Availability

Raw *nirS* sequencing data are available on NCBI under BioProject PRJNA836569. The bioinformatic workflow for sequence analysis and data visualization can be assessed at https://github.com/NancyXiang/nirS-Sequence-Analysis.

## References

[1] Darwin C. The structure and distribution of coral reefs, 3rd edn. D. Appleton & Company: New York, NY, USA, 1889.

[2] Lajeunesse TC, Parkinson JE, Gabrielson PW, Jeong HJ, Reimer JD, Voolstra CR (2018). Systematic Revision of Symbiodiniaceae Highlights the Antiquity and Diversity of Coral Endosymbionts. Curr Biol.

[3] Muscatine L, Porter JW (1977). Reef corals: mutualistic symbioses adapted to nutrient-poor environments. Bioscience..

[4] Rohwer F, Seguritan V, Azam F, Knowlton N (2002). Diversity and distribution of coral-associated bacteria. Mar Ecol Prog Ser.

[5] Rosenberg E, Koren O, Reshef L, Efrony R, Zilber-Rosenberg I (2007). The role of microorganisms in coral health, disease and evolution. Nat Rev Microbiol..

[6] Muscatine L (1990). The role of symbiotic algae in carbon and energy flux in reef corals. Coral Reefs.

[7] Falkowski PG, Dubinsky Z, Muscatine L, McCloskey L (1993). Population control in symbiotic corals. Bioscience..

[8] Baker DM, Freeman CJ, Wong JCY, Fogel ML, Knowlton N (2018). Climate change promotes parasitism in a coral symbiosis. ISME J.

[9] Rädecker N, Pogoreutz C, Gegner HM, Cárdenas A, Perna G, Geißler L (2022). Heat stress reduces the contribution of diazotrophs to coral holobiont nitrogen cycling. ISME J.

[10] Rädecker N, Pogoreutz C, Voolstra CR, Wiedenmann J, Wild C (2015). Nitrogen cycling in corals: the key to understanding holobiont functioning?. Trends Microbiol.

[11] Bourne DG, Webster NS. Coral Reef Bacterial Communities. In: Rosenberg E, DeLong EF, editors. The Prokaryotes. Springer: Berlin Heidelberg; 2013. pp. 163–87.

[12] Ainsworth DT, Krause L, Bridge T, Torda G, Raina J-B, Zakrzewski M (2015). The coral core microbiome identifies rare bacterial taxa as ubiquitous endosymbionts. ISME J.

[13] Pernice M, Raina J-B, Rädecker N, Cárdenas A, Pogoreutz C, Voolstra CR (2020). Down to the bone: the role of overlooked endolithic microbiomes in reef coral health. ISME J.

[14] Pogoreutz C, Oakley CA, Rädecker N, Cárdenas A, Perna G, Xiang N (2022). Coral holobiont cues prime Endozoicomonas for a symbiotic lifestyle. ISME J.

[15] Pogoreutz C, Voolstra CR, Rädecker N, Weis V. The coral holobiont highlights the dependence of cnidarian animal hosts on their associated microbes. In: Bosch TCG, Hadfield MG, editors. Cellular Dialogues in the Holobiont. Boca Raton: CRC Press; 2020. pp. 91–118.

[16] Babbin AR, Tamasi T, Dumit D, Weber L, Rodríguez MVI, Schwartz SL (2021). Discovery and quantification of anaerobic nitrogen metabolisms among oxygenated tropical Cuban stony corals. ISME J.

[17] Glaze TD, Erler DV, Siljanen HMP (2022). Microbially facilitated nitrogen cycling in tropical corals. ISME J.

[18] Lesser MP, Morrow KM, Pankey SM, Noonan SHC (2018). Diazotroph diversity and nitrogen fixation in the coral Stylophora pistillata from the Great Barrier Reef. ISME J.

[19] Cardini U, Bednarz VN, Naumann MS, van Hoytema N, Rix L, Foster RA (2015). Functional significance of dinitrogen fixation in sustaining coral productivity under oligotrophic conditions. Proc R Soc B..

[20] Pogoreutz C, Rädecker N, Cárdenas A, Gärdes A, Wild C, Voolstra CR (2017). Nitrogen fixation aligns with nifH abundance and expression in two coral trophic functional groups. Front Microbiol.

[21] Pogoreutz C, Rädecker N, Cárdenas A, Gärdes A, Voolstra CR, Wild C (2017). Sugar enrichment provides evidence for a role of nitrogen fixation in coral bleaching. Glob Chang Biol.

[22] Bednarz VN, van de Water JA, Rabouille S, Maguer JF, Grover R, Ferrier‐Pagès C (2019). Diazotrophic community and associated dinitrogen fixation within the temperate coral Oculina patagonica. Environ Microbiol.

[23] Lema KA, Willis BL, Bourne DG (2012). Corals form characteristic associations with symbiotic nitrogen-fixing bacteria. Appl Environ Microbiol.

[24] Lema KA, Clode PL, Kilburn MR, Thornton R, Willis BL, Bourne DG (2016). Imaging the uptake of nitrogen-fixing bacteria into larvae of the coral Acropora millepora. ISME J.

[25] Santos HF, Carmo FL, Duarte G, Dini-Andreote F, Castro CB, Rosado AS (2014). Climate change affects key nitrogen-fixing bacterial populations on coral reefs. ISME J.

[26] Rädecker N, Pogoreutz C, Gegner HM, Cárdenas A, Roth F, Bougoure J (2021). Heat stress destabilizes symbiotic nutrient cycling in corals. Proc Natl Acad Sci USA.

[27] Braker G, Fesefeldt A, Witzel K-P (1998). Development of PCR primer systems for amplification of nitrite reductase genes (nirK and nirS) to detect denitrifying bacteria in environmental samples. Appl Environ Microbiol.

[28] Tilstra A, El-Khaled YC, Roth F, Rädecker N, Pogoreutz C, Voolstra CR, et al. Denitrification aligns with N2 fixation in Red Sea corals. Sci Rep. 2019;9:1–9. 10.1038/s41598-019-55408-z PMC6923481 31857601

[29] Tilstra A, Roth F, El-Khaled YC, Pogoreutz C, Rädecker N, Voolstra CR (2021). Relative abundance of nitrogen cycling microbes in coral holobionts reflects environmental nitrate availability. R Soc Open Sci..

[30] Xiang N, Hassenrück C, Pogoreutz C, Rädecker N, Simancas-Giraldo SM, Voolstra CR, et al. Contrasting microbiome dynamics of putative denitrifying bacteria in two octocoral species exposed to dissolved organic carbon (DOC) and warming. Appl Environ Microbiol. 2022;88:e01886-21. 10.1128/AEM.01886-21 PMC8788706 34788073

[31] El-Khaled YC, Roth F, Tilstra A, Rädecker N, Karcher DB, Kürten B (2020). In situ eutrophication stimulates dinitrogen fixation, denitrification, and productivity in Red Sea coral reefs. Mar Ecol Prog Ser.

[32] Beauchamp EG, Trevors JT, Paul JW. Carbon sources for bacterial Denitrification. In: Stewart BA. Advances in Soil Science. Springer: New York, NY; 1989. pp. 113–42.

[33] Baker AC (2003). Flexibility and Specificity in Coral-Algal Symbiosis: Diversity, Ecology, and Biogeography of Symbiodinium. Ann Rev Ecol Evol Syst.

[34] Wang J-T, Chen Y-Y, Tew KS, Meng P-J, Chen CA (2012). Physiological and Biochemical Performances of Menthol-Induced Aposymbiotic Corals. PLoS ONE.

[35] Cui G, Liew YJ, Li Y, Kharbatia N, Zahran NI, Emwas A-H (2019). Host-dependent nitrogen recycling as a mechanism of symbiont control in Aiptasia. PLoS Genet.

[36] Rädecker N, Raina J-B, Pernice M, Perna G, Guagliardo P, Kilburn MR (2018). Using Aiptasia as a Model to Study Metabolic Interactions in Cnidarian-Symbiodinium Symbioses. Front Physiol..

[37] Voolstra CR (2013). A journey into the wild of the cnidarian model systemAiptasiaand its symbionts. Mol Ecol.

[38] Sunagawa S, Wilson EC, Thaler M, Smith ML, Caruso C, Pringle JR (2009). Generation and analysis of transcriptomic resources for a model system on the rise: the sea anemone Aiptasia pallida and its dinoflagellate endosymbiont. BMC Genom.

[39] Xiang T, Hambleton EA, DeNofrio JC, Pringle JR, Grossman AR (2013). Isolation of clonal axenic strains of the symbiotic dinoflagellate Symbiodinium and their growth and host specificity1. J Phycol..

[40] Thornhill DJ, Lewis AM, Wham DC, Lajeunesse TC (2014). Host‐specialist lineages dominate the adaptive radiation of reef coral endosymbionts. Evolution..

[41] Bieri T, Onishi M, Xiang T, Grossman AR, Pringle JR (2016). Relative Contributions of Various Cellular Mechanisms to Loss of Algae during Cnidarian Bleaching. PLoS ONE.

[42] Baumgarten S, Simakov O, Esherick LY, Liew YJ, Lehnert EM, Michell CT (2015). The genome of Aiptasia, a sea anemone model for coral symbiosis. Proc Natl Acad Sci USA.

[43] Correa AMS, McDonald MD, Baker AC (2009). Development of clade-specific Symbiodinium primers for quantitative PCR (qPCR) and their application to detecting clade D symbionts in Caribbean corals. Mar Biol..

[44] Livak KJ, Schmittgen TD (2001). Analysis of relative gene expression data using real-time quantitative PCR and the 2− ΔΔCT method. Methods..

[45] Lee JA, Francis CA (2017). DeepnirSamplicon sequencing of San Francisco Bay sediments enables prediction of geography and environmental conditions from denitrifying community composition. Environ Microbiol.

[46] Huggett J, Dheda K, Bustin S, Zumla A (2005). Real-time RT-PCR normalisation; strategies and considerations. Genes Immun.

[47] Callahan BJ, McMurdie PJ, Rosen MJ, Han AW, Johnson AJA, Holmes SP (2016). DADA2: High-resolution sample inference from Illumina amplicon data. Nat Methods..

[48] Martin M (2011). Cutadapt removes adapter sequences from high-throughput sequencing reads. EMBO J.

[49] Boutet E, Lieberherr D, Tognolli M, Schneider M, Bairoch A. UniProtKB/Swiss-Prot: the manually annotated section of the UniProt KnowledgeBase. Methods Mol Biol. 2007;406:89–112.

[50] Abascal F, Zardoya R, Telford MJ (2010). TranslatorX: multiple alignment of nucleotide sequences guided by amino acid translations. Nucleic Acids Res.

[51] Edgar RC (2004). MUSCLE: multiple sequence alignment with high accuracy and high throughput. Nucleic Acids Res.

[52] Kearse M, Moir R, Wilson A, Stones-Havas S, Cheung M, Sturrock S (2012). Geneious Basic: An integrated and extendable desktop software platform for the organization and analysis of sequence data. Bioinformatics..

[53] Fish JA, Chai B, Wang Q, Sun Y, Brown CT, Tiedje JM (2013). FunGene: the functional gene pipeline and repository. Front Microbiol.

[54] Wickham H. ggplot2. Wiley Interdiscip Rev Comput Stat. 2011;3:180–5.

[55] Oksanen J, Kindt R, Legendre P, O’Hara B, Stevens MHH, Oksanen MJ (2007). The vegan package. Commun Ecol Package.

[56] McMurdie PJ, Holmes S (2013). phyloseq: An R Package for Reproducible Interactive Analysis and Graphics of Microbiome Census Data. PLoS ONE.

[57] Lin H, Peddada SD (2020). Analysis of compositions of microbiomes with bias correction. Nat Commun..

[58] Meunier V, Geissler L, Bonnet S, Rädecker N, Perna G, Grosso O (2021). Microbes support enhanced nitrogen requirements of coral holobionts in a high CO 2 environment. Mol Ecol.

[59] Geissler L, Meunier V, Rädecker N, Perna G, Rodolfo-Metalpa R, Houlbrèque F, et al. Highly Variable and Non-complex Diazotroph Communities in Corals From Ambient and High CO2 Environments. Front Mar Sci. 2021;8:754682.

[60] Thornhill DJ, Xiang Y, Pettay DT, Zhong M, Santos SR (2013). Population genetic data of a model symbiotic cnidarian system reveal remarkable symbiotic specificity and vectored introductions across ocean basins. Mol Ecol.

[61] Röthig T, Costa RM, Simona F, Baumgarten S, Torres AF, Radhakrishnan A (2016). Distinct bacterial communities associated with the coral model Aiptasia in aposymbiotic and symbiotic states with Symbiodinium. Front Mar Sci.

[62] Hartman LM, Blackall LL, van Oppen MJH (2022). Antibiotics reduce bacterial load in Exaiptasia diaphana, but biofilms hinder its development as a gnotobiotic coral model. Access Microbiol.

[63] Lawson CA, Raina JB, Kahlke T, Seymour JR, Suggett DJ (2018). Defining the core microbiome of the symbiotic dinoflagellate, Symbiodinium. Environ Microbiol Rep..

[64] Matthews JL, Raina JB, Kahlke T, Seymour JR, van Oppen MJ, Suggett DJ (2020). Symbiodiniaceae‐bacteria interactions: rethinking metabolite exchange in reef‐building corals as multi‐partner metabolic networks. Environ Microbiol..

[65] Costa RM, Cárdenas A, Loussert-Fonta C, Toullec G, Meibom A, Voolstra CR. Surface Topography, Bacterial Carrying Capacity, and the Prospect of Microbiome Manipulation in the Sea Anemone Coral Model Aiptasia. Front Microbiol. 2021;12:637834. 10.3389/fmicb.2021.637834 PMC8060496 33897642

[66] Pelve EA, Fontanez KM, DeLong EF (2017). Bacterial succession on sinking particles in the ocean’s interior. Front Microbiol..

[67] Welles L, Lopez-Vazquez CM, Hooijmans CM, Van Loosdrecht MCM, Brdjanovic D. Prevalence of ‘Candidatus Accumulibacter phosphatis’ type II under phosphate limiting conditions. AMB Express. 2016;6:1–12. 10.1186/s13568-016-0214-z PMC4932009 27376945

[68] Kaneko T (2002). Complete Genomic Sequence of Nitrogen-fixing Symbiotic Bacterium Bradyrhizobium japonicum USDA110. DNA Res.

[69] Cziesielski MJ, Liew YJ, Cui G, Schmidt-Roach S, Campana S, Marondedze C (2018). Multi-omics analysis of thermal stress response in a zooxanthellate cnidarian reveals the importance of associating with thermotolerant symbionts. Proc R Soc B: Biol Sci..

[70] Xiang T, Lehnert E, Jinkerson RE, Clowez S, Kim RG, Denofrio JC (2020). Symbiont population control by host-symbiont metabolic interaction in Symbiodiniaceae-cnidarian associations. Nat Commun..

