# Supplementary material for: Presence of algal symbionts affects denitrifying bacterial communities in the sea anemone Aiptasia coral model
Source: ISME Commun. 2022 Oct 28;2:105. doi: 10.1038/s43705-022-00190-9 (PMC9723753; doi:10.1038/s43705-022-00190-9)
Supplement: Supplementary file 1 — Supplementary Information [file 43705_2022_190_MOESM1_ESM.pdf]

Supplementary information

Supplementary Fig. S1

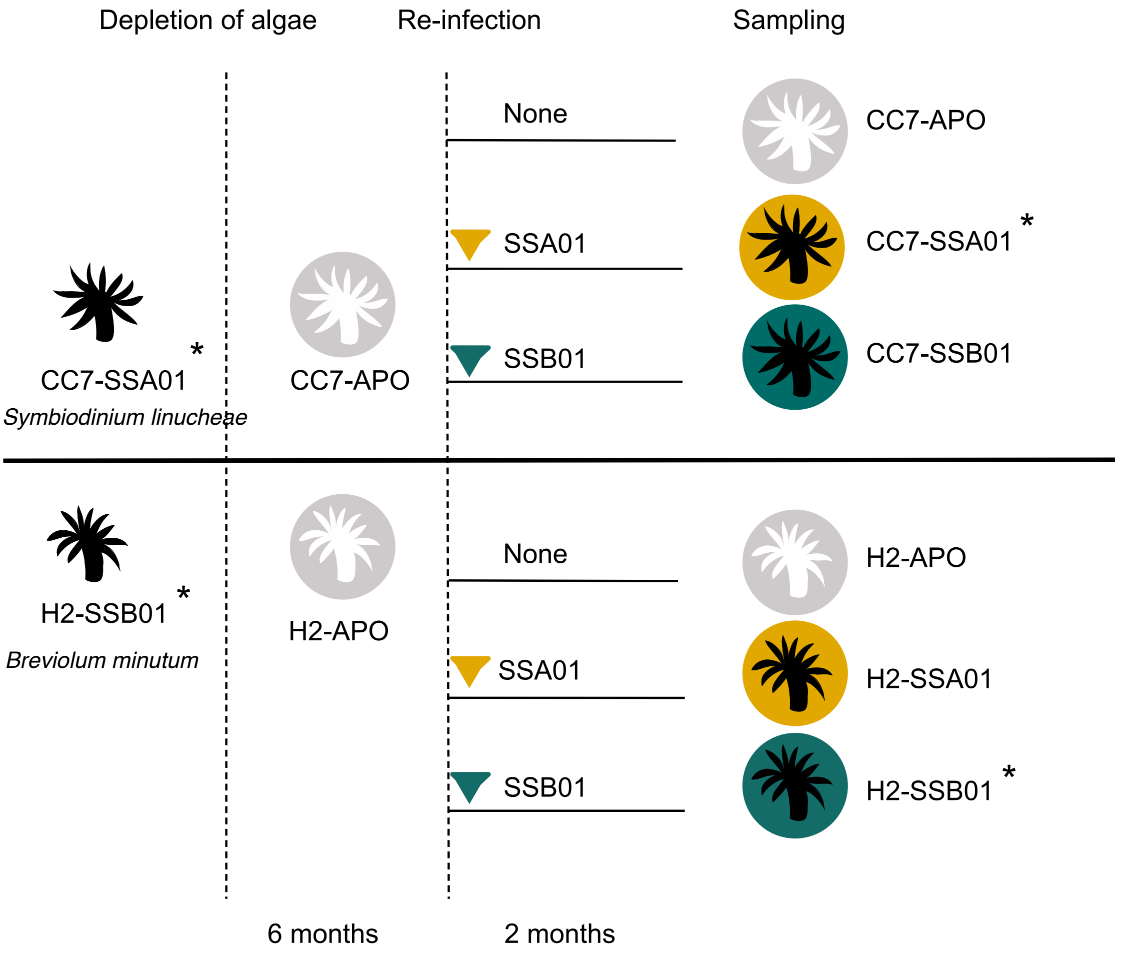

Supplementary Fig. S1 Overview of aposymbiotic Aiptasia generation, algal inoculations and sampling. Aiptasia strains CC7 and H2 were inoculated with two strains of Symbiodiniaceae: *Symbiodinium linucheae* strain SSA01 and *Breviolum minutum* strain SSB01. The native host-symbiont combinations of Aiptasia are indicated by asterisks (*).

Supplementary Fig. S2

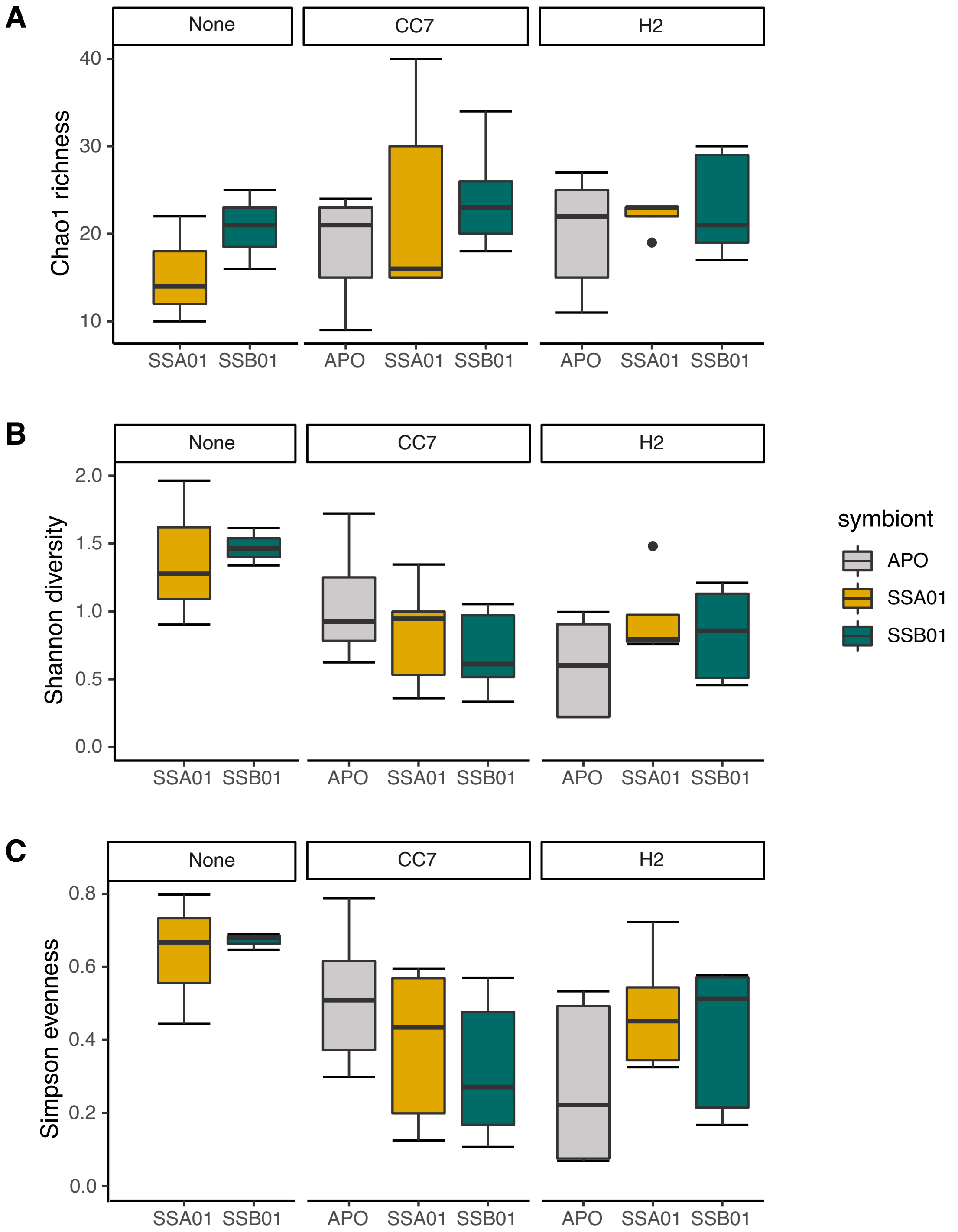

Supplementary Fig. S2 Alpha diversity of denitrifier communities in Aiptasia animals, Symbiodiniaceae, and food samples based on *nirS* gene sequencing. **A** Chao1 richness. **B** Shannon index. **C** Simpson evenness.
